# Conference report WHO informal consultation on the draft WHO Guideline on the phasing out of animal tests for the quality control of biological products

**DOI:** 10.1016/j.biologicals.2025.101862

**Published:** 2025-11

**Authors:** Ian Feavers, Dianliang Lei, Catherine Milne, Ivana Knezevic, Tiequn Zhou, Eunkyung Kim

**Affiliations:** a, Nacton, Suffolk, UK; b Unit of Technical Specifications and Standards, Department of Health Product Policy and Standards, Division of Access to Medicines and Health Products, World Health Organization, Geneva, Switzerland; c European Directorate for the Quality of Medicines and HealthCare (EDQM), Council of Europe, Strasbourg, France

**Keywords:** WHO Guideline, Animal test, Replacement, Removal, Quality control, Biological products

## Abstract

Animal testing has long supported the development and quality control of biotherapeutics and vaccines by ensuring safety and efficacy. However, its variability and time-consuming nature can delay product availability. Advances in non-animal technologies, guided by the 3Rs principles, have led to more efficient and scientifically robust alternatives. Recognizing the limitations of animal assays, WHO encourages their replacement when scientifically justified and has drafted a Guideline on phasing out animal tests in biological product quality control. Following public consultation, an informal meeting at WHO Headquarters brought together regulators, industry representatives, and other stakeholders to review the draft. The Guideline was developed based on ECBS recommendations and a review of existing WHO documents. Participants proposed improvements, including a revised title, to better emphasize the scientific rationale for replacing animal-based tests used in quality control scheme. These updates aim to support finalization of the document for a second public consultation and ECBS adoption.

## Highlights

• The World Health Organization (WHO) has a long-standing and vital role in developing guidelines and recommendations for the production, quality control, and nonclinical and clinical evaluation of biological products. To support regulators and manufacturers, WHO developed a guideline focused on the removal and replacement of animal tests in the quality control of biological products.
• An informal consultation was held at WHO Headquarters, bringing together participants from national regulatory authorities, manufacturers, and other stakeholders to review the draft guideline, which had already undergone public consultation. Taking into account the feedback received, the participants reviewed the draft text and proposed improvements to assist the drafting group in finalizing the document for submission to the Expert Committee on Biological Standardization (ECBS) for adoption.
• Publishing the outcomes of this consultation will help stakeholders and the public understand the development of this important guideline and its implications for advancing science and ethical practices in biological product testing.

## 1 Introduction

**The World Health Organization (WHO) provides leadership in global health matters with the principal objective of attaining the highest possible level of health for everyone. In this regard, one of its core functions is setting norms and standards, and promoting their implementation.** WHO has a long-established and important role in developing guidelines and recommendations (written standards) on the production, quality control, nonclinical and clinical evaluation of biological products. Globally these norms and standards, based on scientific consensus achieved through extensive international collaboration and consultation, support WHO Member States in ensuring the quality, safety and efficacy of biological products. WHO written standards, which are reviewed and recommended for adoption by the WHO Expert Committee on Biological Standardization, aim to keep abreast of technological advances and be consistent with other regulatory guidance worldwide. They are published in the WHO Technical Report Series (TRS).

Historically, animal models have played an important part in the development of biological medicinal products, providing critical information on their mechanisms of action, safety and efficacy. In some cases, however, these methods continue to be used post-approval to monitor product quality and/or safety as part of the quality control processes carried out by manufacturers and national control laboratories. Driven by scientific and technological developments, the application of 3Rs principles, and recognition that *in vitro* approaches are typically superior to existing animal tests for routine quality control, there has been significant progress in the development and implementation of non-animal methods for testing biological products [[1], [2], [3], [4], [5]]. Increasingly, these developments are being incorporated into national and regional regulatory guidelines (e.g. Ph.Eur. 5.2.14) [6].

Recognizing the importance of these developments and the need for WHO guidance to be aligned with current thinking amongst manufacturers and regulators globally, in 2019 ECBS endorsed a WHO proposal to undertake an independent review of animal testing requirements in its existing guidelines and recommendations. The review was carried out by the UK National Centre for the Replacement, Refinement and Reduction of Animals in Research (NC3Rs) and co-funded by the Bill and Melinda Gates Foundation [[7], [8], [9]]. It identified animal tests recommended for quality control, noting where alternative strategies were already proposed and where opportunities for incorporating non-animal tests or barriers exist. In critical areas of *in vivo* quality control testing, the text of WHO guidance was also reviewed and alternative, standardized language proposed to harmonize future revisions. The key recommendations from the review included the need for: a) a WHO position statement and guidance on the incorporation of 3Rs practices into lot release testing functions based on sound scientific principles; b) a manual on pyrogenicity and endotoxin testing; and c) improvements to the accessibility and utility of WHO guidance.

Following presentation of the outcome of the review to the ECBS in October 2023 and acknowledging the challenge of revising many existing WHO guidelines quickly, ECBS recommended the establishment of a working group consisting of national regulators and independent experts to develop science-based guidance on the removal and replacement of animal tests used in the quality control of biological products [9]. Reflecting on the current status of animal testing in the quality control of biologicals, the recommendations of the NC3Rs review and requests from industry (i.e. IFPMA and DCVMN) and other stakeholders (i.e. EDQM, EFPIA, NC3Rs, EPAA and IABS), the working group prepared a concept paper outlining the structure of a prospective WHO Guideline on the phasing out of animal tests for the quality control of biological products and setting out the timeline for its development. A drafting group consisting of eleven experienced regulators then prepared a first draft of the proposed guideline, which was revised in light of comments received from a public consultation. An informal consultation was held in Geneva, Switzerland, between 27 and 28 February, 2025 to review current practices and regulations in animal testing amongst participating countries, and to consider outstanding issues and potential improvements to the revised text. In addition to the drafting group and WHO staff, the informal consultation sought the expert advice of participants from national regulatory authorities (NRAs), the biopharmaceutical industry and a range of other stakeholders.

## 2 Update on biological standardization activities at WHO

To provide meeting participants with context for the current draft guideline, Dr Knezevic, Team Lead of WHO Norms and Standards for Biologicals and Secretary of the Expert Committee on Biological Standardization, gave an update on the current status of WHO standards for biologicals. She explained that the WHO norms and standards team is responsible for 113 written standards, published in the WHO Technical Report Series (TRS), and more than 500 measurement standards, which are essential for the development and quality control of biologicals. Highlighting the outcomes of the 80th meeting of ECBS, held in October 2024, she reviewed the written standards that had recently been recommended for adoption by ECBS and updated participants on several new or revised guidelines that were under consideration from 2024 onwards. This included the draft Guideline on phasing out of animal test for the quality control of biological products, which is expected to be submitted to ECBS in October 2025. She outlined the role of WHO Collaborating Centres for biological standardization in developing written and measurement standards, as well as supporting implementation workshops, noting that three (MHRA, PEI, CBER) as well as EDQM served as custodian laboratories for measurement standards. She concluded by summarizing updates and improvements to the WHO catalogue and highlighting key WHO events on biological standardization in 2025.

Responding to a question on the prospects for expediting the revision of WHO Guidelines and Recommendations that currently promote the use of animal-based quality control tests, Dr Knezevic explained that this is a complex issue that will not be resolved quickly; however, WHO staff were exploring innovative ways to highlight such changes on the WHO website.

Dr Knezevic mentioned a long history of WHO activities towards rational use of animals for testing potency and safety of vaccines and other biologicals [10,11]. She also provided brief explanation of the importance of the progress with high throughput sequences (HTP), also known as next generation sequencing, as a regulatory tool for reducing and replacing animal testing in the evaluation of biologicals which has been elaborated in the meeting reports from the conferences organized by the International Association on Biological Standardization (IABS) [[12], [13], [14]].

Dr. Lei Responsible officer of the project, Scientist of WHO Norm and Standards of Biologicals gave a presentation explaining the background of using animal testing in the development, manufacture, and quality control of vaccines and other biological products, as well as WHO's position on the application of the 3Rs principles in routine testing and releasing of biological products. He reminded the participants of the scientific limitations of many animal assays and emphasized that WHO encourages manufacturers and regulators to adopt the 3Rs principles and move away from animal-based research, production, and quality control whenever scientifically justified. He also reiterated that validated *in vitro* alternatives should be favored wherever possible, and the type of testing should be driven by the scientific need and valid relevant data.

At its 2023 meeting, the ECBS recommended establishing a working group to develop WHO science-based guidance encouraging the replacement of animal tests with non-animal tests in the quality control of vaccines and other biological products [9]. Following this recommendation, a drafting group was set up with members from regulatory authorities to initiate the work. Meanwhile, a concept paper addressing the current challenges and regulatory practices in using non-animal tests was developed to guide the group in proceeding with the process. An outline of the guidelines and steps for development was proposed in the concept paper. Based on this the group drafted the first draft of Guideline on the phasing out of animal tests in the quality control of biological products and ready for the review by the meeting.

Dr. Lei finally outlined the objectives of the meeting, which were to review current practices and regulations in animal testing in participating countries and revise the draft Guideline on the phasing out of animal tests in the quality control of biological products.

He introduced Dr Catherine Milne (EDQM) who was appointed as the chair of the meeting to facilitate the discussions and foster consensus.

## 3 Overview of 3Rs principles in QC and lot release of vaccines, and current practices of manufacturers and regulators

### 3.1 WHO perspective

Dr Ian Feavers, WHO consultant gave a presentation on overview of 3Rs principles in quality control and lot release of vaccines. He outlined that animal testing has a long-established role in the development of biological products; however, these tests tend to become embedded in the routine quality control testing schemes of approved products. For the quality control of biologicals, test methods are intended to monitor product consistency and ensure commercial lots are comparable with those demonstrated to be safe and efficacious in clinical studies. Nevertheless, there is a considerable body of evidence that animal tests are inherently variable making them poorly suited for this purpose and highlighting the need to use *in vitro* alternatives wherever possible.

Nowadays, the quality control of biological products must be considered in the context of significant advances in production processes and laboratory technologies. The application of good manufacturing practices (GMP) ensures consistent production and conformance to quality standards, while quality management systems (QMS) document processes, procedures and responsibilities to reduce mistakes and support continual improvement. Quality by design (QbD) approaches, which have been part of WHO and ICH guidance since 2009, ensure that quality is built into a product and that the critical quality attributes underpinning control testing schemes are clearly defined. Technological advances in molecular, physicochemical and cell-based methods provide a wealth of opportunities for the development of *in vitro* quality control tests, and many recently developed products do not require animal tests (e.g. polysaccharide conjugate and mRNA vaccines).

He reminded participants that the purpose of WHO Guidelines is to provide key principles for the evaluation of biological products as a basis for setting national or regional requirements, whilst at the same time allowing space for National Regulatory Authorities (NRAs) to include additional or more specific requirements. They are intended to be developed further or revised in line with scientific developments and experience, and consider guidance issued by other organizations, so as to complement rather conflict with them. The WHO position on the use of animals in the quality control of biological products is articulated in the WHO Guideline on lot release [15], which stresses the potential variability of *in vivo* tests and the need to apply 3Rs principles (reduction, replacement, refinement) for ethical reasons. It suggests that, driven by scientific need, validated *in vitro* alternatives should be favored as far as possible and that National Control Laboratories (NCLs) should establish mutual recognition or collaborative agreements to share the results of *in vivo* tests and minimize animal testing worldwide. More recently, WHO has published clear statements on the discontinuation of discredited innocuity testing (i.e. general safety or abnormal toxicity test), as well as supporting the implementation of molecular alternatives to neurovirulence and adventitious agent testing [16,17].

### 3.2 European regulatory perspective

Dr Catherine Milne described the work of the European Directorate for the Quality of Medicines and HealthCare (EDQM) to implement strategies that remove and replace animal testing in Europe. She started by explaining the status of EDQM as a Directorate of the Council of Europe and its contribution to public health through ensuring access to good quality medicines. Setting the context for EDQM's work on 3Rs, she went on to describe the overarching legal framework in Europe for the protection of animals used in science and how this is implemented through the European Pharmacopoeia (Ph. Eur.), where general chapters and individual monographs, together with general notices, encourage reduction in animal usage and promote the application of 3Rs principles. Ph. Eur. texts are subject to continual review to ensure they are consistent with current scientific opinion on the use of 3Rs and the removal of obsolete methods. The main areas of 3Rs activity at EDQM include: Ph. Eur., the Biological Standardisation Programme (BSP) and the Official Medicines Control Laboratories' (OMCLs) Network. The application of 3Rs for vaccines and potentially other biological products is facilitated by chapter 5.2.14 of Ph. Eur. [6], which provides guidance on introducing alternative *in vitro* methods where a head-to-head comparison is inappropriate and presents the concept of substitution as an alternative to replacement. It focuses on the scientific rationale supporting the use of *in vitro* methods.

Dr Milne continued by summarizing some of EDQM's notable achievements and future opportunities. These included removal of the abnormal toxicity test from Ph. Eur. in 2017 and the rabbit pyrogen test from Ph. Eur. texts from July 2025, which, with the removal of animal tests for histamine and depressor substances from January 2026, will effectively have abolished all general animal safety tests from the Ph. Eur. within the next year. In addition, the application of 3Rs principles to pharmacopoeial texts has led to the rationalization of toxicity tests used in assessing the safety of DTaP combination vaccines. Recognizing the potential for HTS to replace animal tests for adventitious agents, a new Ph. Eur. chapter (2.6.41) was under development for adoption in March 2025 [6].

The BSP has made an important contribution to the implementation and acceptance of 3Rs methods exemplified by work to: support the inclusion of the BINACLE *in vitro* test for tetanus toxicity in tetanus vaccine monographs; develop an *in vitro* potency assay for human rabies vaccine; and examine the feasibility of a cell-based assay to measure the potency of recombinant erythropoietin *in vitro*. Playing a major part in these developments, OMCLs are often early implementers of 3Rs opportunities and promote the use of alternative methods through training and sharing experience. Furthermore, European Official Control Authority Batch Release reduces the use of animals through a legal obligation for mutual recognition, where the test results of one Official Medicines Control Laboratory are accepted by all member states.

Dr Milne concluded by summarizing challenges for the implementation of *in vitro* alternative methods for the quality control of biological products. These challenges include: a) the need for accessible methods that are not restricted by intellectual property rights or the availability of critical reagents; b) the time required to develop, validate and implement tests; c) the universal acceptance of *in vitro* approaches; and d) specification setting, where the focus on consistency makes decisions a matter between the manufacturer and individual NRAs, increasing the prospect of divergence between different authorities.

### 3.3 Indonesian regulatory perspective

Presenting the perspective of the National Quality Control Laboratory for Drug and Food (NQCLDF), Mr Dio Ramondrana described the status of 3Rs implementation in Indonesia. He explained that the criteria for regulatory acceptance of alternative *in vitro* approaches depended on the availability of scientifically relevant, well-defined methods with demonstrable reliability and robustness. In Indonesia, 3Rs implementation is based on national guidelines, including the Indonesian Pharmacopoeia and its regulation on preclinical toxicity testing, as well as international guidelines such as the WHO TRS, the US Pharmacopoeia and EMA guidance on the regulatory acceptance of 3Rs approaches. Indonesia continues to make important progress towards the implementation of *in vitro* testing for biological products including the removal of the requirement for abnormal toxicity testing, the validation of an *in vitro* potency test for recombinant COVID-19 vaccine and the development of the monocyte activation test (MAT) as an alternative to the rabbit pyrogenicity test (RPT). In addition, NQCLDF is supporting the development of HTS as a replacement for the monkey neurovirulence test (MNVT) and has taken steps to reduce the number of animals used to test the potency of tetanus and hepatitis B vaccines. Refining other methods, NQCLDF has introduced humane endpoints for challenge assays and improvements that lower the severity of other tests. It has also introduced a range of improvements to the environment and welfare of laboratory animals.

Mr Ramondrana went on to set out the Indonesian view of the challenges and opportunities presented by the implementation of 3Rs methods for quality control testing. Challenges include harmonizing international regulatory guidance; establishing the comparability of *in vitro* tests with the so-called “gold standard” animal tests; the commitment required to transition from *in vivo* to *in vitro* methods; and ensuring a similar level of competence between the regulator and industry. Acknowledging the better reproducibility of *in vitro* tests, he noted that the opportunities potentially included: quicker, cheaper product release testing; fewer ethical and animal welfare concerns; positive support from regulators and manufacturers; and the creation of an environment where everyone recognizes the scientific and economic advantages of adopting non-animal methods. He concluded by highlighting the Indonesian FDA's support for the development of the WHO Guideline, highlighting the importance of scientific evidence that alternative methods will consistently ensure product quality and of the commitment from all stakeholders to invest in the implementation of *in vitro* test methods.

### 3.4 Japanese regulatory perspective

Dr Koji Ishii of the National Institute of Infectious Diseases (NIID) described progress towards the replacement of animal testing for the quality control of biological products in Japan. He started by summarizing the status of animal tests performed on vaccines, highlighting that the abnormal toxicity test had been removed from testing schemes for all products by March 2023. There has also been important progress towards the replacement of animal tests for two specific vaccines. For rabies vaccines, changes to potency tests include the introduction of humane endpoints and development of an *in vitro* assay for assessing vaccine inactivation. For Japanese encephalitis vaccine, where potency and inactivation are assessed using a mouse model, NIID is also currently considering the introduction of an antigen ELISA method to replace the existing potency test.

Dr Masaaki Iwaki then went on to explain difficulties encountered with replacing detoxification tests or potency tests based on challenge with tetanus or diphtheria toxins, citing the subjectivity of scoring tetanus toxicity and the frequent failure to determine a humane endpoint as particular challenges. He presented the results of studies that support the transition from a lethal challenge to an ELISA-based methodology for tetanus toxoid potency testing and from a rabbit intradermal test to a Vero cell neutralization test for diphtheria toxoid potency. He concluded by expressing concerns about not performing toxicity reversion tests, highlighting a fatal outcome associated with the administration of reverted toxoid. Although meeting participants broadly agreed that poor quality toxoids might revert under certain circumstances, they questioned whether this had ever been observed with GMP-produced lots and why an *in vitro* alternative could not be used.

### 3.5 Canadian perspective

Drs Tong Wu and Julie Joseph provided meeting participants with the view of Health Canada on the use of animal-based tests for the quality control of biological products. Employing a risk-based approach, Health Canada's lot release programme takes into account the nature of the product, the target population, its own experience of lot release of the product and the manufacturer's production and testing history. As a result, testing focuses on value added tests that assess key quality attributes for products considered to be of higher risk. The tests used are manufacturer adapted, compendial or independently established methods. Despite the absence of 3Rs legislation, Health Canada has a long-established history of supporting the use of non-animal tests for the quality control of biological products and its decisions on replacing animal-based tests are typically driven by scientific considerations rather than 3Rs principles. Health Canada has seen a steady decline in the number of animal-based tests during the last two decades and, since 2022, none have been performed for routine lot release. It also strongly supports and encourages manufacturers to replace *in vivo* tests with *in vitro* alternatives wherever scientifically justified.

Reflecting on their expectations for the proposed WHO Guideline, Health Canada expected the WHO to provide strong, science-based guidance endorsing the replacement of *in vivo* methods used for quality control with fit-for-purpose *in vitro* alternatives. Emphasizing that sound scientific considerations should be the principal driver for replacement of animal-based tests, Health Canada recognized that reduction and refinement approaches still require animals and are inferior to replacement with fit-for-purpose and validated alternatives.

### 3.6 International Federation of Pharmaceutical Manufacturers and Association (IFPMA)

Drs Emmanuelle Coppens and Sarah Sheridan briefed meeting participants on the pharmaceutical industry's perspective on the use of animals in the quality control of biological products and their thoughts on the draft WHO Guideline. For investigational products, current practice is only to use animals for preclinical studies, relying on thorough product characterization and use of the most appropriate *in vitro* technologies as the basis of quality control testing. Legacy products, however, present a greater challenge as they are generally poorly characterized and testing for safety and efficacy has largely depended on *in vivo* assays. For these products, industry has focused on strategies to switch to more scientifically relevant and reliable *in vitro* tests. Examples include removal of scientifically unjustified safety tests such as the innocuity test; replacement of *in vivo* potency assays by immunoassays, cell-based assays or physicochemical assays; replacement of RPT with MAT or the bacterial endotoxin test (BET); and replacement of *in vivo* adventitious agents testing with molecular sequencing and cell-based assays.

They highlighted some of the challenges the pharmaceutical industry faces when trying to implement innovative *in vitro* approaches to quality control testing. The lack of global harmonization for safety and potency testing presents a particular problem, with significant regional and national requirements resulting in double testing for the manufacturer and/or redundant testing by the NCL, which increases the risk of out of specification and invalid results. They argued that post approval changes to introduce alternative approaches represent a high regulatory burden, typically requiring a strong rationale and data package to convince NRAs. In addition, despite the guidance on the substitution approach provided by Ph. Eur. 5.2.14, many NRAs continue to expect head-to-head comparison of innovative *in vitro* tests with existing animal-based methods, even when scientifically unjustified.

The IFPMA made several helpful comments on the draft WHO Guideline, suggesting that the sections proposing alternatives to animal tests should be more detailed and appropriate, and consistent use of terminology would ensure clarity. Noting the superiority of *in vitro* methods for quality control schemes, they highlighted the importance of QbD approaches and GMP to ensure consistency, rather than relying on animal-based quality control tests to demonstrate compliance. They also emphasized the importance of regulatory reliance mechanisms and harmonization in reducing the burden of testing.

Meeting participants pointed out that legacy products have the advantage of a consistency profile established over many years, which could be used to facilitate the transition from *in vivo* to *in vitro* methods. Noting that its Guideline for post-approval changes for vaccines and biotherapeutic products were currently being revised, WHO felt by taking a risk-based approach and reinforcing reliance mechanisms the revised Guideline would help to reduce the regulatory burden associated with the process.

### 3.7 Developing country Vaccine Manufacturers Network (DCVMN)

Representing DCVMN, Dr Pradip Kumar Das provided the meeting with the vaccine industry's perspective on the phasing out of animal tests from quality control schemes. Focusing on the importance of quality control throughout vaccine production, he highlighted that animals continue to be widely used for research and development, in process control and lot release. Although quality control is based on demonstrating consistency of production using analytical methods, the complexity of vaccines currently requires extensive testing of each lot, with safety and potency often being assessed in animals. Acknowledging the large number of animals used to ensure the safety and potency of vaccines, he emphasized the importance of 3Rs principles and the implementation of *in vitro* alternatives to these tests going forward.

Addressing the traditional potency tests for whole-cell pertussis, rabies, tetanus and diphtheria vaccines, which depend on animal challenge methods, he reviewed various reduction and refinement approaches that have been implemented including the use of single- rather than multi-dilution assays and cell-based methodologies. Historically, specific toxicity testing has been crucial for ensuring the safety of toxin or toxoid-containing vaccines such as those for diphtheria, tetanus and pertussis as well as polysaccharide conjugates. Nowadays, analytical methods that quantify the protective antigen obviate the use of animal potency tests for more recently developed vaccines. DCVMN members accept and are working to implement the key changes to specific toxicity testing set out in WHO Guidelines and manuals, with superior cell-based assays increasingly replacing *in vivo* specific toxicity tests for diphtheria and pertussis vaccines. In addition, demonstration of consistency of production may be used to justify the removal of the test from routine lot release. They also recognize the importance of refining and ultimately replacing other safety tests including those for neurovirulence, adventitious agents and pyrogens.

Like the IFPMA, DCVMN highlighted the lack of harmonization of regulatory requirements globally, the complexity of post-approval changes and the reluctance of some NRAs to accept deviations from established guidelines as major regulatory hurdles to the implementation of 3Rs approaches. In addition, the inherent variability of *in vivo* assays and the fact that product quality attributes are likely to be assessed differently were recognized as key scientific challenges when switching from *in vivo* to *in vitro* methods. Dr Das concluded by summarizing DCVMN's expectations for the WHO Guideline and specific comments on the draft text.

### 3.8 UK National Centre for the 3Rs (NC3Rs)

Dr Elliot Lilley described the role of NC3Rs in supporting 3Rs approaches in testing biological products. Noting that NC3Rs works with industry, academia, regulators and funders worldwide, he outlined its remit and strategy, which is based on delivering scientifically driven, evidence-based outputs. He went on to set out NC3Rs position on animal use in lot release testing. Globally, as many as 10 million animals are used each year in the development, production and quality control of biological products, of which at least two-thirds are used in routine lot release tests for licensed products. These tests are expensive, can cause significant pain and distress to the animals and tend to be highly variable, potentially causing lengthy delays to the release of some products. The NC3Rs advocates transitioning away from animal testing for lot release where non-animal technologies are scientifically relevant and appropriately validated. Where animals continue to be used, NC3Rs advocates optimising experimental design to reduce animal numbers and employing high standards of animal housing, husbandry and care to maximise animal welfare.

Dr Lilley then summarized the objectives and outcomes of the NC3Rs review of animal use in WHO Guidelines and Recommendations, including the recommendations listed in its report, which had been submitted to ECBS in October 2023 [8]. These included: a table of all animal tests in WHO guidance, with recommended alternative language incorporating 3Rs methods; a proposal for standalone guidance on pyrogenicity testing; a proposal for a general 3Rs statement from WHO; and recommendations on the stewardship of the WHO TRS. A subsequent survey of stakeholders had highlighted some of the challenges associated with the implementation of 3Rs approaches in quality control testing but recognized that integration of 3Rs in WHO Guidelines and Recommendations would have a positive impact on regulatory acceptance globally, providing NRAs with confidence in supporting the replacement of animal-based tests. The survey identified that the leadership WHO provides in the global harmonization of regulatory approaches, written standards and training could have a significant impact on the adoption of 3Rs methods for the quality control of biological products.

Dr Lilley concluded by summarizing NC3Rs expectations for WHO, noting there is a clear consensus amongst stakeholders that, in most cases, non-animal methods are superior to existing animal-based methods for quality control, being scientifically more relevant and less variable. Given WHO's leadership in this area and the respect for WHO written standards, he underlined the importance of WHO as a driver for positive change and commended the draft guideline as broadly following the recommendations of the NC3Rs review of WHO Guidelines and Recommendations.

## 4 Review comments received from public consultation and draft WHO guideline

Having reviewed the current practices and views of biological product manufacturers and regulators, the informal consultation meeting went on to review the second draft of the proposed *WHO Guideline on the phasing out of animal tests used in the quality control and lot release of biological products* considering comments received from the first-round public consultation. Thirty-one organizations had responded to the first-round public consultation including national and regional regulators, biological products manufacturers, manufacturers of kits and reagents, manufacturers’ networks, and ethical societies. This had resulted in more than 500 comments ranging from suggestions for minor edits to major changes; however, noting the considerable redundancy amongst the comments, the drafting group had rationalized the key issues to be addressed in a revised draft. In addition to numerous comments on specific sections of document, the drafting group had received several general comments on the document as a whole, including putting more emphasis on the scientific justification for *in vitro* tests used in the quality control of biological products rather than ethical considerations, harmonizing the layout of the specific considerations sections and recognizing the challenge of implementing the Guideline once adopted, as well as suggestions for alternative titles.

Based on this feedback, the drafting group had produced a second draft of the proposed Guideline for consideration by participants attending the informal consultation. There was a consensus amongst the drafting group that, given scientific and technological advances, the time was right to end the use of animal-based tests for the quality control of biological products. Consequently, the revised draft placed more emphasis on the scientific justification for the replacement and complete removal of *in vivo* tests, whilst encouraging the development of quality control schemes for new products that avoid the use of animals from the outset. The structure of this draft was consistent with other similar Guidelines published in the WHO TRS, consisting of an introduction, purpose and scope, general considerations, specific considerations, reference materials and guidance for regulators. The specific considerations were further subdivided to address six key areas where *in vivo* testing continues to be used in quality control tests for: innocuity, adventitious agents, pyrogens, neurovirulence, potency and specific toxicity.

Overall, participants of the informal consultation felt that the document had been significantly improved by changes made following the first-round public consultation. Reviewing the draft in detail, they discussed a wide range of issues and suggested numerous potential edits. The key discussions and proposed changes to the draft document were as follows:

### 4.1 Title

Reflecting on comments raised by the first-round public consultation, meeting participants discussed the proposed title and agreed to replace “… phasing out …” with “… replacement or removal …” which they felt was more consistent with the second draft's stronger focus on ending the use of animals in quality control testing schemes and its emphasis on replacement rather than refinement or reduction approaches. Moreover, this modification addressed concerns of some that the phrase “phasing out” lacked urgency, implying that animal testing might be removed over a long timeframe. The agreed title of the third draft would, therefore, be, “*Guidelines on replacement or removal of animal tests for the quality control of biological products*.”

### 4.2 Revision of WHO Guidelines and Recommendations

The public consultation had raised the question of how the document would address the revision of existing WHO Guidelines and Recommendations with the product- or test-specific texts recommended. Both WHO and the drafting group agreed this was beyond the scope of the draft Guideline but that going forward there needed to be a discussion about the implementation of 3Rs for legacy products and the revision of their WHO Guidelines and Recommendations. Dr Knezevic indicated that WHO was discussing possible interim solutions utilizing the WHO website.

Due to the challenges associated with swiftly revising documents published in the WHO TRS, there was a strong consensus that there should be a clear statement, highlighted in a text box early in the document, that the implementation of *in vitro* strategies for the quality control of biological products detailed in this document should not await revision of existing WHO Guidelines and Recommendations. As such, the proposed new Guideline would effectively supersede existing guidance regarding *in vivo* quality control tests.

### 4.3 Introduction

As the focus of the second draft of the proposed Guideline was on the scientific justification for quality control schemes based on *in vitro* methods, the informal consultation participants discussed whether references to the 3Rs in the introduction should be removed entirely. The drafting group felt that, as the intention of the introduction was primarily to provide an historical perspective and explain the evolution of the document, the 3Rs should not be removed altogether. Instead, the introduction was edited to make clear the historical role of 3Rs in underpinning work towards the removal of animal tests.

### 4.4 Purpose and scope

Comments received from the first-round public consultation suggested that there was a lack of clarity on where the existing guidelines were still to be considered applicable. The informal consultation agreed that a paragraph should be added to make it clear that this Guideline takes precedence when implementing quality control requirements concerning animal-based tests specified in existing WHO Guidelines and Recommendations. However, to ensure its prominence, it was agreed that this statement should be in a text box at the start of the document (see “Revision of WHO Guidelines and Recommendations” above).

### 4.5 Terminology

The language for the entries describing replacement, reduction and refinement was edited to be consistent with the definitions currently provided by NC3Rs whilst retaining their relevance to product testing. In addition, the term “Viscerotropism” was added.

### 4.6 General considerations

Several comments received from the first-round public consultation suggested that the proposed Guideline should focus on the scientific advantages of *in vitro* tests rather than the 3Rs. In light of these suggestions, this section was completely revised to focus on the superiority of and scientific justification for *in vitro* methods in quality control schemes. The emphasis on scientific arguments to support the use of *in vitro* methods has been integrated throughout the text, while still acknowledging the ethical importance of reducing animal testing. In addition, the revised text is more aligned with Ph. Eur. 5.2.14 in supporting the removal or replacement of animal-based tests rather than focusing on 3Rs.

Acknowledging that animals continue to be used for regulatory testing of biological products, this section included a paragraph to highlight the importance of animal welfare. The informal consultation agreed that this was an important issue that needed to be retained in the document but would be strengthened by citing open, non-regional references. Following the informal consultation, Dr Elliot Lilley (NC3Rs) provided an alternative paragraph and references to be included in the next draft of the document.

### 4.7 Innocuity

Reflecting on the order of the specific consideration sections, the informal consultation participants agreed that innocuity testing (i.e. the general safety or abnormal toxicity test) should be the last sub-section. The rationale was that WHO had already made clear public statements that innocuity testing should not be performed and there was evidence that manufacturers and regulators were working towards removal of the test from quality control schemes. Furthermore, the sections on adventitious agent, pyrogen and neurovirulence testing, where there has been good progress towards the implementation of *in vitro* alternatives, would be more prominent if innocuity testing were moved to the end of the section.

There was also a strong consensus that the proposed Guideline should include an appendix listing current WHO documents for biological products published prior to 2018 where the innocuity test should be disregarded.

### 4.8 Adventitious agents

Because HTS and other molecular methods do not distinguish between viable and non-viable agents, there was a proposal from the first-round public consultation to use *in vivo* tests to confirm the presence of adventitious agents in the event of a positive result from a molecular test. Participants at the informal consultation agreed that the proposal was inappropriate and the existing wording should be retained but also include a reference to Ph. Eur. 2.6.41.

They also supported clarification of the text to ensure it was aligned with ICH Q5A (R2) guidance that *in vivo* adventitious virus assays and antibody production tests can be replaced with molecular methods including polymerase chain reaction (PCR) and HTS with targeted or non-targeted detection.

### 4.9 Endotoxin and pyrogenicity testing

The informal consultation proposed several minor changes to the text of the second draft of the document for clarity, including: defining what is meant by “product specific reference”; noting that the readout of the MAT is based on cytokines with a role in fever pathogenesis; and removing specific examples of cell lines used in MAT.

### 4.10 Neurovirulence

Regarding neurovirulence tests performed for the quality control of yellow fever vaccines, there was a discussion about whether to include a paragraph on the quality control of new products based on yellow fever vectors; however, it was agreed that this should be covered as part of preclinical development and would be out of the scope of this document. In addition, several minor changes to the text were made for clarity.

### 4.11 *Transition* from *in vivo to in vitro potency testing*

This section was broadly revised in line with discussions at the informal consultation meeting. Minor edits clarified the potential use of out-of-specification lots as test samples to ensure alternative *in vitro* potency assays can distinguish meaningful changes in composition and quality and also highlighted that antibodies used for vaccine antigen detection need to be well-characterized. In addition, it was agreed the conclusions sub-section should note the important role of manufacturers in driving change through multi-stakeholder engagement recognizing the important influence of the Vac2Vac project.

### 4.12 Specific toxicity

For diphtheria, tetanus and acellular pertussis vaccine components, current WHO recommendations include tests for reversion to toxicity. Following extensive discussion, a consensus was reached that the proposed WHO Guideline should recommend that routine testing of lots for reversion to toxicity is not necessary if the detoxification process has been validated to the satisfaction of the NRA. This would ensure better alignment with the guidance of national and regional authorities that no longer require a routine test for reversion.

Regarding specific toxicity testing for whole cell pertussis vaccine, a comment from the first-round public consultation questioned whether an *in vitro* alternative had been developed; however, unaware of such a development, the participants of informal consultation agreed to alter the text for clarity by reversing the word order to read, “… develop and use a validated *in vitro* assay …”.

There was also a brief discussion raising a general concern that more sensitive *in vitro* tests may fail product lots, which would otherwise pass a less sensitive *in vivo* test, and it was agreed that this issue should be captured in the general considerations.

### 4.13 Development and use of international and other reference materials

Acknowledging that in certain cases animal methods may continue to be necessary for calibration of secondary standards, participants of the informal consultation discussed the specific example where heterogeneity in glycosylation of therapeutic protein hormones can result in the relationship between the *in vivo* test and *in vitro* alternative being product specific. Although the *in vitro* assay may be suitable for routine lot control, the link to *in vivo* bioactivity and product dosing would require calibration of a secondary standard *in vivo*. This would be included in the third draft of the proposed Guideline.

### 4.14 Other changes

In addition to the changes outlined above and the re-ordering of the specific considerations sections, participants recommended that each section should end with a summary of its conclusions in a sub-section entitled “Conclusions” rather than “Recommendations”.

They also agreed that the proposed Guideline should reinforce that *in vivo* tests should not be regarded as a gold standard and that out-of-specification results or other questionable outcomes obtained with *in vitro* alternatives should not initiate repeat tests in animals, nor should animal tests be the fall-back for assessing manufacturing changes. A statement to this effect would be added to the general considerations of the next draft of the document.

### 4.15 Implementation

Noting the slow implementation worldwide of WHO advice not to use the obsolete innocuity test, the informal consultation discussed possible solutions to support the implementation of this Guideline once it was adopted. Participants were reminded that WHO Guidelines provide a basis for setting national and regional requirements but are not legally binding. Participants accepted that as WHO TRS documents are revised, they will be changed to conform with this Guideline; however, this will take time. They, therefore, considered more immediate approaches to ensure that this Guideline results in changes to pharmacopoeias and hence regulatory practice, and leads to greater international harmonization so that animal tests are not performed to satisfy jurisdictions that are not aligned with its guidance.

In addition to workshops and online materials typically employed to implement WHO Guidelines and Recommendations, participants suggested several other approaches. Recognizing the pivotal role of WHO prequalification and regulatory strengthening teams in the implementation of WHO Guidelines, participants highlighted how the engagement of these teams with a range of other agencies might be used to leverage specific expertise and support. They also suggested WHO might consider publishing a set of frequently asked questions (FAQs) online once the Guideline is adopted. Participants reiterated the importance of focusing on the scientific justification for using *in vitro* tests for quality control rather than the ethical considerations, when implementing the Guideline.

## 5 Closure of the informal consultation

Before the closure of the meeting, Dr Dianliang Lei set out the next steps for the development of the Guideline. By the end of March, a third draft of the proposed Guideline would be prepared by the drafting group based on the discussions at the informal consultation and circulated to participants for further comments. A final revision of the third draft would then be sent for editing from May, prior to a final public consultation and submission to ECBS for its recommendation on adoption in the third quarter of 2025. He concluded by thanking participants for their commitment in reviewing the document.

Dr Catherine Milne concluded the meeting by thanking participants for their time and commitment, noting the high level of engagement from all throughout the meeting.

## 6 Conclusions, recommendations to WHO

In summary, there was a consensus that the WHO Guideline should focus on the replacement or removal of animal tests for the quality control of biological products guideline. Such a guideline is crucial to regulators, biological product industry and other related organizations. The proposed revision and suggestions by the meeting participants and comments from the first-round public consultation would be considered by the drafting group and used to revise the text of the guideline. The finalized guideline will be subject to a further round of public consultation and revision before being submitted to ECBS for review at its meeting October 2025.

## CRediT authorship contribution statement

**Ian Feavers:** Writing – original draft. **Dianliang Lei:** Writing – original draft, Project administration. **Catherine Milne:** Writing – original draft. **Ivana Knezevic:** Writing – review & editing. **Tiequn Zhou:** Writing – review & editing. **Eunkyung Kim:** Writing – review & editing.
